# Are Humans Prepared to Detect, Fear, and Avoid Snakes? The Mismatch Between Laboratory and Ecological Evidence

**DOI:** 10.3389/fpsyg.2019.02094

**Published:** 2019-09-11

**Authors:** Carlos M. Coelho, Panrapee Suttiwan, Abul M. Faiz, Fernando Ferreira-Santos, Andras N. Zsido

**Affiliations:** ^1^Department of Psychology, Faculty of Psychology, Chulalongkorn University, Bangkok, Thailand; ^2^Department of Psychology, Dev Care Foundation, Dhaka, Bangladesh; ^3^Department of Psychology, Laboratory of Neuropsychophysiology, Faculty of Psychology and Education Sciences, University of Porto, Porto, Portugal; ^4^Department of General and Evolutionary Psychology, Institute of Psychology, University of Pécs, Pécs, Hungary

**Keywords:** general feature detection, modular theories, snake bite kinematics, selective habituation hypothesis, evolutionary psychology

## Abstract

Since Seligman (1971) statement that the vast majority of phobias are about objects essential to the survival of a species, a multitude of laboratory studies followed, supporting the finding that humans learn to fear and detect snakes (and other animals) faster than other stimuli. Most of these studies used schematic drawings, images, or pictures of snakes, and only a small amount of fieldwork in naturalistic environments was done. We address fear preparedness theories and automatic fast detection data from mainstream laboratory data and compare it with ethobehavioral information relative to snakes, predator-prey interaction, and snakes’ defensive kinematics strikes in order to analyze their potential matching. From this analysis, four main findings arose, namely that (1) snakebites occur when people are very close to the snake and are unaware or unable to escape the bite; (2) human visual detection and escape response is slow compared to the speed of snake strikes; (3) in natural environments, snake experts are often unable to see snakes existing nearby; (4) animate objects in general capture more attention over other stimuli and dangerous, but recent objects in evolutionary terms are also able to be detected fast. The issues mentioned above pose several challenges to evolutionary psychology-based theories expecting to find special-purpose neural modules. The older selective habituation hypothesis (Schleidt, 1961) that prey animals start with a rather general predator image from which specific harmless cues are removed by habituation might deserve reconsideration.

## Introduction

Worldwide, yearly snakebites leading to death could be as high as 94,000 (Kasturiratne et al., 2008) or even more according to the World Health Organization. The annual number of fatalities can be as high as 125,000, with an additional 400,000 amputations and other severe health consequences (World Health Organization, 2013). Considering these numbers, one may argue that snakes to date pose a real threat to human survival, and thus, a specific defense system might have been adaptive throughout the course of primate evolution. Indeed, many studies have shown that humans and other primates might have an attention bias toward evolutionary threats such as snakes. Some researchers (Öhman and Mineka, 2003; Isbell, 2006) consider that humans might have a *dedicated* “threat-detection” or “snake detection” *module* that favors both faster detection and fear learning of material related to snakes.

The present manuscript collects information about snake behavior and kinematics as well as epidemiological information from countries where snakebites are more frequent or better described. Our aim is twofold: (1) describe the circumstances snakebites usually take place and (2) find out whether this information can help us support or refute the general assumption that humans have a unique ability to detect snakes, even under camouflage conditions, due to a specific snake defensive mechanism.

## Modular Theories Relative to Snakes

What are the salient features of dangerous objects that are missing in similar, but harmless ones? Since 1937 experiments are done, intending to know how we are able to discern what a life-threatening animal looks like (see Schleidt et al., 2011). Seligman (1971) proposed that a vast majority of phobias would relate to particular objects and events essential to the survival of the human species. Seligman’s paper prompted scientists to test his theory, using pictures of snakes and other potentially harmful animals (Öhman et al., 1974). This line of research later culminated in a theory that humans have a *perceptual apparatus* to efficiently detect snakes and other threats (Öhman and Mineka, 2003), even under challenging visual conditions (Soares et al., 2014; Kawai and He, 2016) due to the evolutionary value of this ability (see Soares et al., 2017 for a review).

LeDoux suggested the possibility of a “visual bypass” (LeDoux, 1998) to convey rapid visual information to the amygdala and protect humans from, for instance, snakes. This was based on an earlier finding (LeDoux et al., 1990) that the auditory cortex is not required for a sound to be paired with a shock during fear conditioning, as projections from the acoustic thalamus are sent directly to the amygdala. However, the visual bypass is less sharp as it does not benefit from cortical processing. As such it is a crude representation of the stimulus, LeDoux calls it a “*quick and dirty processing pathway*” (LeDoux, 1998, p. 164) that could lead to a number of false positives discoveries. LeDoux (1998) and others (see e.g., Flykt, 2006; LoBue, 2014) argued that it is nonetheless better to err on the side of caution than to wait until the cortex figures out whether the object is a snake or a curved stick.

Not much later, it was further shown that humans could discriminate visual stimuli that are not consciously perceived, due to connectivity between the right amygdala and the superior colliculus and the pulvinar nucleus of the thalamus (Morris et al., 1999). This circuit became known as the *low road*, a route able to “avoid” a longer cortical route since it goes directly to the amygdala from the superior colliculus and the pulvinar nucleus of the thalamus. The combined findings gave additional vivacity to a theory of fear, based on a low road, and many researchers use this idea to explain their results. Moreoverd, additional research supported that humans could respond to masked stimuli of snakes (Öhman and Soares, 1994) with an enhanced physiological fear response. The recognition of the snake stimulus (presented for 30 ms) had been prevented by an immediately following masking stimulus, presented for 100 ms. Still, participants with a fear of snakes showed enhanced skin conductance to both the masked and the unmasked pictures of snakes (presented for 130 ms), although they were not able to consciously identify the masked ones.

The low road gave additional vivacity to theories of fear (but cf., Pessoa and Adolphs, 2010) suggesting the existence of a *quick visual pathway* designed to facilitate detection of a threat as a first signal that something dangerous might be present. Thus, preparing the person to deal with a potential hazard even before it reaches awareness. In support of this view, Van Le et al. (2013) showed that the pulvinar response latency to snake pictures was shorter (within 50 ms post-stimulus onset) and faster than response latencies to other visual stimuli, such as faces and geometrical shapes, and was associated with a significant increase in gamma band oscillations in pulvinar neurons (Van Le et al., 2016).

This previous body of work specifies that a subcortical visual route operates independently of the cortex, having a role in pre-attentive affective processing, by providing a rapid direct pathway to the amygdala for snake-detection. Indeed, it has been argued that snakes seem to elicit fast behavioral responses, facilitating rapid detection, focused attention, and avoidance in humans and other primates (Maior et al., 2011; Soares, 2012; Van Le et al., 2016). Overall, several theories favor that humans have a set of prewired responses embedded in an evolved brain circuit or dedicated modular system built up over phylogeny which allows for faster detection of snakes, comparatively to most other stimuli. This idea feeds from a more general impression that the brain is composed of highly selective and functionally specialized areas connected along developmentally and evolutionarily dedicated pathways (see Anderson, 2016; Badcock et al., 2016). But some data are inconsistent with the modular theories, as we will address next.

## Data Incompatible With Modular Theories

1. Considering that it is better to err on the side of caution than to wait until the cortex figures out whether the seen object is a snake or a curved stick, people should be afraid of all kinds of snakes or snake-like animals and objects. Indeed, it has been suggested (Larson et al., 2007, 2009, 2012; Van Strien et al., 2016) that curved lines and *curvilinear shapes* have an advantage in visual processing. Respondents seem faster to find a curved line as targets among straight lines compared to when they had to find a straight line among curved ones. Furthermore, curvilinear shapes are also detected faster than rectilinear (V-shaped) shapes when they had to be found among the same set of distractors, i.e., straight lines or circles.
  False alarms (e.g., looks like a snake but after all it is a log) are less costly than false negatives (e.g., looks like a log but it is a snake); thus, it is safer to err on the side of excessive defensive expression (see e.g., LeDoux, 1998; Nesse, 2001). However, if the system is built to be over-defensive and err to the side of false positives, it should respond not only to snake-specific cues but also to other similar threat-related stimuli because many non-venomous snakes mimic venomous snakes, and venomous snakes exhibit extreme pattern variability and different characteristics (e.g., Wüster, 1998). For example, young white-faced capuchin monkeys (*Cebus capucinus*) are known to utter false alarm calls at a wide range of harmless non-predator animals (e.g., indigo snakes) compared to adults, and snake-species discrimination does not become apparent until the juvenile stage (Meno et al., 2013). Similarly studies with vervet monkeys (*Chlorocebus pygerythrus*) (Seyfarth and Cheney, 1980) and with spectral tarsiers (*Tarsius spectrum*) (Gursky, 2003) found youngsters to provide false alarm calls triggered by a wide range of harmless animals or objects, suggesting that this predator-recognition process likely involves experiential and vicariant learning refinements (Meno et al., 2013). Another example is given by the forest-living Campbell’s monkeys (*Cercopithecus C. campbelli*) that vocalized to the presence of familiar Gaboon vipers but not to unfamiliar black mambas (*Dendroaspis polylepis*), unreported in the Taï forest where the study was performed (Ouattara et al., 2009).
2. Previous research has shown (see e.g. Subra et al., 2018; Zsido et al., 2018a) using various paradigms that a *modern threatening* stimulus could lead to similar behavior as an evolutionary relevant one. For instance, when modern (e.g., gun) and evolutionary (e.g., snake) targets are compared directly in the classical visual search task proposed by Öhman and colleagues (see e.g., Öhman et al., 2001), the modern threatening target caught participants attention faster than evolutionary ones (Zsido et al., 2018b).
3. *Low-level features* of the visual stimulus could have affected many of the previous findings, as contrast/luminance and spatial frequency affect visual detection speed and processing of emotional content during early visual stages (Vlamings et al., 2009; Quinlan, 2013). Most studies did not control for low-level features of the visual stimulus (including contrast) of snakes, which can be significantly different from those of other categories and can affect the findings. These effects of contrast equalization on spatial frequency processing are particularly crucial during early (i.e., < 100 ms) visual processing (McFadyen et al., 2017).
4. In tropical and subtropical countries, envenoming affects mainly people involved in occupations and lifestyles requiring movements in a dense land, such as farmers, herders, labourers, hunters, shepherds, and workers (Meenatchisundaram and Michael, 2009). However, most of the previous work relied on controlled laboratory paradigms without support from studies in *naturalistic conditions*, which may be the cause of many sorts of potential bias (Quinlan, 2013; Paré and Quirk, 2017).
5. Many snakes pigmentation pattern appears to be strongly influenced by selection to avoid visually oriented predators (Jackson et al., 1976). Snakes rely on *crypsis* – (i.e., background matching) to become indistinguishable from the surrounding background (Isaac and Gregory, 2013) as one of many strategies to avoid detection (see Allen et al., 2013).
  So even highly experienced observers with over 20 years’ experience miss seeing most snakes (more than 75%) aboveground around them in a flat and lightly vegetated area (Whitaker and Shine, 1999a,b). However, this could also suggest that snakes developed crypsis since the primate visual system was so effective to detect snakes.
6. Most snakebites follow at very close vicinity (Whitaker et al., 2000; Clark et al., 2012) and are extremely fast (Cundall, 2002; LaDuc, 2002; Clark et al., 2012; Penning et al., 2016), outside the capability for humans to escape.
7. There is evidence supporting that the human right amygdala is a specialized neural adaptation dedicated to processing visual information about animals in general (Mormann et al., 2011), which could represent either predators or prey, such as snakes. Indeed, humans do kill and eat snakes in several cultures (Headland and Greene, 2011). It seems likely that human’s enhanced detection of snakes is not so much contributing toward a significant escape advantage, but it might instead favor attention and further evaluation (Purkis and Lipp, 2007). When a stimulus needs to be approached in one situation may be avoided in another (Mesulam, 1998), the brain is more adapted to *behavioral flexibility* than to automatic, modular responses. In the case of snakes, the evidence seems to point to a similar conclusion.
8. Various laboratory studies comparing snakes and spiders (animate objects) as prepared stimuli with flowers and mushrooms might have been flawed by *animacy* as a confound. Flowers and mushrooms “behave” similarly to inanimate objects (e.g., coffee mugs and telephones). Change-detection, for instance, is slower and less precise for inanimate targets, even when the inanimate targets can potentially move (e.g., tools or cars) or have evolutionary relevance such as plants or fruits (New et al., 2010; Jackson and Calvillo, 2013). This evidence does not necessarily deny the snake detection theory, but previous papers using flowers and mushrooms are often used and cited to support the snake detection theory (e.g., Soares et al., 2017).
9. Some of the characteristics of human-snake interaction do not appear to be the ones commonly seen between *prey-predator interactions* that lead to specific evolved automated traits. Ultra-specializations make sense for ultra-dependent predator-prey interactions, such as the kangaroo rat—rattlesnake interaction. These rodents escape speed may have evolved in response to snake predators (Higham et al., 2017), as Mohave rattlesnakes (*Crotalus scutulatus*) show very fast accelerations (up to 362 ms^−2^) requiring from the kangaroo rats a response around 61.5 ms in order to escape. Although humans evolved the large cortex that could modify instinct behaviors, these take time and reduce the automaticity and response speed.
10. Finally, Grassini et al. (2016) found that snakes need to be consciously perceived to elicit emotion. Furthermore, it was also shown that affective processing requires *awareness* (Lähteenmäki et al., 2015).

The abovementioned discussion points raise the question of why and how humans would develop an ultra-fast detection mechanism specific to snakes. In the next part of the paper, we present some information about snakes that hopefully can assist in disentangling this problem.

## Snakes and Snakebites

A few species of snakes are responsible for most fatal snakebites. The most reported are the (1) Krait (*Bungarus caeruleus*); (2) Saw-scaled viper (*Echis carinatus*); (3) Russell’s viper (*Daboia russelii*); (4) Cobra–Spectacled Cobra (*Naja naja*), and Monocled Cobra (*Naja kaouthia*) (e.g., Punde, 2005; Bawaskar et al., 2008) which will be next briefly described as examples. Some information from Rattlesnakes (*Crotalus*) kinematics, Eastern Brownsnake (*Pseudonaja textilis*), and Cottonmouths (*Agkistrodon piscivorous*) crypsis are also addressed. Other particular species are punctually mentioned when relevant.

### Common Krait (*Bungarus caeruleus*)

The krait is a nocturnally active snake that lives close to human dwellings (Deshwal and Gupta, 2015). Although usually not aggressive, it often creeps into farmer’s habitations to feed on rodents (Bawaskar et al., 2004). This leads bites during involuntary sleep movement (Ariaratnam et al., 2008) while snakes benefit from people’s body heat (Bawaskar and Bawaskar, 2015). This particular interaction suggests that this snake is likely unrelated to any selective pressure to primates’ evolution of some form of visual detection bias.

### Russell’s Viper (*Daboia russelii*)

Russell’s viper is distributed throughout Southeast Asia (Pathan et al., 2015). This snake is usually about 120 cm and typically does not strike unless provoked, before which it hisses a loud warning (Odedara, 2007). However, its bite presents high morbidity and mortality rates (Alirol et al., 2010; Sharma et al., 2014). Russell’s viper is nocturnal, and bites occur during the early day and at dusk, affecting farmers in agricultural lands usually when in paddy fields, clearing bushes, cutting grass in home gardens, walking unprotected on narrow footpaths, growing grass or jungle tracks (Kularatne, 2003; Kularatne et al., 2011, 2014). The bite mostly happens when the snake is stepped on (Ariaratnam et al., 2009). Interestingly, when people intentionally search for snakes, such as field herpetologists and collectors, they need to look under rocks, logs, boards, sheet metal, and debris, as well as collapsed buildings, where refuge places abound to be able to find these reptiles (Fitzgerald, 2012) as snakes are not easy to spot.

The Russell’s viper snakebite characteristics might make it a candidate to have been an evolutionary selective pressure. It appears to be a snake challenging to discover and additionally it emits a warning sound before striking. It would be important to know if people that are bitten were previously “warned” by the snake and if they are able to detect them in the abovementioned terrains.

### The Indian Cobras, Spectacled Cobra (*Naja naja*), and Monocled Cobra (*Naja kaouthia*)

The cobras of the genus *Naja* are distributed throughout most Asian countries and usually range from 100 to 150 cm. Cobras usually feed on rodents, frogs, toads, birds, lizards, other snakes, and eggs (Wüster, 1998). They can be found in termite nests, holes in dams of rice fields, stonework constructions, and tree base roots. The monocled cobra is also a significant life-threatening source but not aggressive, always attempting to escape and offering plenty of warning before biting even when cornered (Wüster, 1998). The fact that bites occur during daytime and dusk hours and include areas of human development (toilets, houses, etc.) and natural areas (bushes, trenches, water reservoirs, etc) (Rahman et al., 2010; Bawaskar and Bawaskar, 2015) suggests that these are being defensive bites that occur when both the human and the snake were not “expecting” each other.

### Saw-Scaled Viper (*Echis carinatus*)

The Saw-scaled Viper size is responsible for the most significant number of snakebite cases (Warrell, 1995): usually, less than 45 cm long, very cryptic, bites under small provocation (Odedara, 2007) very quickly, averaging 69 ms (20–108 ms) (Cundall, 2017, personal communication). This snake often bites farmers, hunters, labourers, and persons walking barefoot in the jungle and rocky areas. Often the victim interprets the injury as being caused by a thorn, failing to notice the bite (Bawaskar and Bawaskar, 2015).

The saw-scaled viper snakebite characteristics make it a strong candidate to have been a potential selective pressure responsible for the development of a particular detection module in the primate visual system. Many people do not see the snake in time to avoid being bitten but one can argue: “imagine how many venomous snakes would bite even more if we did not have this ability?” To better understand this problem, it would be essential to collect information about how many strikes were avoided and in what conditions. How many snakes people are able to see and avoid comparatively to other potentially dangerous animals?

### Crypsis

Snakes are cryptic animals usually immobile if buried and fleeing if at the surface (Maritz, 2012). Whitaker and Shine (1999a) recorded the responses of free-ranging Brown snakes to 455 close encounters with humans. They implanted miniature radio transmitters in 40 snakes in agricultural landscapes area and found that in about half of the encounters the snake retreated or relied on crypsis. Moreover, snakes advanced offensively toward the observer on only three occasions. Similarly, in a study (Gibbons and Dorcas, 2002) with Cottonmouths, most snakes tried to escape when confronted.

We next address some relevant information about snakebites concerning their strike kinematics and later compare it to the human response reaction times.

### Snake Strike

Functional morphology studies on snake strike kinematics often divide the strike into four phases namely: (1) preparation, (2) extension, (3) contact, and (4) release (Kardong and Bels, 1998). Strike kinematics divided into different stages (Kardong and Smith, 2002), but here we collected information considering the method of division in four phases.

The viperid strike is probably the best studied, usually starting from a coiled position and rapidly straightening the forefront of the body to carry the head toward the prey (Kardong and Bels, 1998). Laboratory data on strikes reveal a typically predatory strike range of 3–20 cm and extension phases between 20 and 50 ms (Clark et al., 2012). A recent study (Penning et al., 2016) revealed strike extension phase durations between 48 and 84 ms in distances between 8.6 and 27.0 cm in several snake species, and western diamond-backed rattlesnakes (*Crotalus atrox*) accelerations could reach 279 ms^−2^. Another *in situ* studies (Clark et al., 2012) observed 37 predatory extension recordings, which revealed extension phase durations between 20 and 100 ms in distances between 5 and 50 cm, in several snake species of *Crotalus* (rattlesnakes). Successful strikes typically started from a distance less than 15 cm of the prey. A study from Whitaker et al. (2000) with the Brown snake shows an overall mean strike duration of 0.28 s (SD = 0.12) and a strike mean distance of 34 cm, between 12 and 64 cm in defensive strikes toward approaching humans. A study from Araújo and Martins (2007) regarding defensive strikes in five species of lanceheads of the genus *Bothrops* (Viperidae) revealed average strike distances of about 10 cm and a strike duration of about 100 ms, with *B. alternatus* being the fastest, 80.6 ms (66.7–110.0).

In sum, most snake bites occur in very *close proximity* to the prey, typically from 3 to 20 cm, and defensive strikes up to 50 cm (Clark et al., 2012) or 60 cm (Whitaker et al., 2000). Furthermore, they are *extremely quick*, usually below 100 ms, both in predatory (Cundall, 2002; Clark et al., 2012) and defensive modes (LaDuc, 2002; Penning et al., 2016). Overall, the data show very variable values, but still very fast compared to human reaction times, as we will see next. These velocity values also suggest that the detection advantage needs to happen when the human is still far enough from the snake to have time to avoid it, and this distance should be more than 60 cm, or about “one step” away from the snake.

## Human Response Speed to Detect and Avoid Snakes

Amygdala modulation of responses by the affective content of stimuli was observed to start at around 200 ms (Krolak-Salmon et al., 2004), although quick *amygdala responses* can begin as early as 74 ms post-stimulus onset (Méndez-Bértolo et al., 2016). Humans could show autonomic responses in response to unconscious fearful objects through a subcortical fear module that could respond in very short latencies (Silverstein and Ingvar, 2015). Although strong enough evidence is lacking, some previous studies showed prefrontal response to emotional stimuli in less than 100 ms post-stimulus onset (see e.g., Carretié et al., 2004; Rehbein et al., 2014). The actual duration of *visual processing* itself is likely to be around 100–150 ms post-stimulus (Thorpe et al., 1996; VanRullen and Thorpe, 2001; Liu et al., 2009). However, the *response time* to a snake (e.g., pressing a key) image takes about 400 ms (Haberkamp et al., 2013), a result similar to the one reported by Thorpe et al. (1996) for general image categorization. *Search times* to find a snake among other distractor stimuli take nearly 1,000 ms (Öhman et al., 2001). Note that, for example, the findings of Flykt and colleagues (e.g., Flykt and Caldara, 2006; Flykt et al., 2012, 2017) do not support an involvement of early information processing in the detection of the feared animal in fearful participants, they favor that short response time to biological threats are mostly due, not to fast detection, but to motor preparations to threats. In sum, they find that a more elaborate analysis of these complex stimuli is required to achieve the detection of the feared animal in fearful participants (Flykt and Caldara, 2006); that focused attention can lead to increases in manual force (Flykt et al., 2012); and that they provide no support for the notion that fear responses can be triggered by stimuli presented outside awareness (Flykt et al., 2017).

More recently, Van Strien et al. (2016) followed similar standard procedures for visual search tasks but comparing simple curvilinear with rectilinear shapes (among straight line distractors). Search times were now reduced compared to the previous more complex figures (Öhman et al., 2001) and further reduced after fear induction, but still around 600 ms. The results can be interpreted in terms of response to threatening stimuli. However, the search times do not seem to translate in a quick-enough response to bring any significant advantage when dealing at close proximity with snakes. As we have seen, the entire snake strike sequence often occurs in less than 100 ms. Moreover, the abovementioned laboratory studies were mainly conducted using hand reaction times, which travel a shorter route compared to lower extremity limbs which exhibit delayed reaction times (Pfister et al., 2014) and is where snakes most often bite (e.g., Reid, 1975; Currie et al., 1991; Hansdak et al., 1998).

A recent work showing monkeys are responsive to visual cues of snake scales (Isbell and Etting, 2017) is one of the few ecologically valid studies so far. These results support the claim that vervet monkeys skillfully detect and remember snakes in the wild. This experiment was partially replicated with humans (Van Strien and Isbell, 2017) but again only in laboratory conditions.

## Non-Modular Theories

The possible existence of a fear module (Öhman and Mineka, 2003) for snake detection ought to be validated (or refuted) in an ecologically valid context. In a natural environment, detecting the shared characteristic of many potential predators and natural dangers such as a looming motion, scales, curves or sharpness, makes perfect sense. Many other animals with scales and fangs that slither or crawl can cause damage and infections, such as some lizards, spiders, scorpions, centipedes, lepidopteran larvae, and gastropods. Consequently, a wide range of innovative structures has evolved to facilitate the delivery of venoms, such as “*barbs, beaks, fangs modified teeth, harpoons, nematocysts, pincers, proboscises, spines, sprays, spurs, and stingers*” (Casewell et al., 2013, p. 219). Considering the multitude of threat sources, it seems to be more useful to have a *general preparedness* (see e.g., Davey, 1995; Coelho and Purkis, 2009; Zsido et al., 2018a,b,c) to detect and respond to animate or sharp objects in general. Evolving a module for each of these animals or features would be impractical. Furthermore, it would also risk that the evolutionary time to build up a particular brain module could very well take longer than the existence of this potential predator, due to other selective pressures.

The *animate monitoring hypothesis* suggests that animate objects in general capture more attention—for their significance ever since human ancestral environments—over other stimuli (New et al., 2007; Nairne et al., 2013; Altman et al., 2016; Calvillo and Hawkins, 2016). Thus, providing another explanation to why humans might detect snakes quickly in laboratory tasks. However, this theory is not able to explain why, for example, people can detect guns faster (e.g., Zsido et al., 2018b) or why snakes are detected faster than birds (e.g., Van Strien and Isbell, 2017).

The *selective habituation hypothesis* (Schleidt, 1961) predicts that prey animals start with a rather general predator image from which specific harmless cues are removed by habituation. Form an evolutionary point of view, a neural mechanism for fast detection of threats would capture only general potential harms (e.g., simple curvilinear shapes) that could signal the presence or a threat, instead of particular and unique snake-like features, and then become specialized with experience. This model predicts that initially animals will be pre-programmed to be alert and scared of many stimuli. Then, this preparedness will generate costs from false alarms but not from missed detections. It does not require experience with the predator since any unusual cue that falls within a certain predator class elicits a response. But by selective habituation–learning what not to fear–potential preys pursue a more advantageous strategy (Deecke et al., 2002). Similarly, for example, the innate avoidance of coral snakes by birds extends to other toxic or harmless snakes and caterpillars (Janzen et al., 2010). Although it has only been shown with domestic chicks, there is evidence that innate fear responses might reset if enough time elapses between encounters with snakes or snake-like animals (Skelhorn et al., 2015).

The data and results collected in the present review favor the selective habituation hypothesis (Schleidt, 1961), i.e., prey animals start out with a rather general predator image from which specific harmless cues are removed by habituation. These models also match with Rachman (2002) suggestion that people *gradually obtain* the required abilities to deal with their current predisposed fears through habituation and experience. The environment can work, therefore, toward eliminating biologically relevant fears, which are likely not specific (e.g., snake), but more general (e.g., looming, scales, or sharpness) and according to context.

## Conclusion

The present study gathered information from (1) snake behavior and strike acceleration, (2) bite circumstances, (3) crypsis; (4) human visual speed processing for detection of complex figures, (5) human reaction time when responding to images of snakes, as well as the fact that (6) snakes are common prey to humans. These are still challenges to the fear module theory or any theory that favors snakes detection advantage, resulting in an expressive way, in successful avoiding venomous snakebites or contributing with a significant escape advantage.

It seems likely that an innate ability exists to respond to simpler features, such as relative speed, size, and contrast of moving objects, before discrimination between different kinds of snakes or snakelike objects. If so, infants might have a predisposition to pay attention to everything sharp and become more specialized over time and experience. Animals and humans may become frightened by many stimuli with different physical, yet similar other characteristics that are not species-specific but rather associated with the context of predation, e.g., movement, intensity, duration, suddenness, or proximity (Boissy, 1995; Davey, 1995; Coelho and Purkis, 2009). Predation in primate populations includes 176 species of confirmed or potential predators of primates, from birds to mammals, reptiles, and sharks (Hart and Sussman, 2005). As such, it seems unlikely that humans have a special brain module for detection of a particular species. Moreover, many non-venomous snakes look like noxious ones, and many venomous snakes exhibit extreme pattern variability and different characteristics (e.g., Wüster, 1998). This led us to doubt the hypothesis that there is a proneness to detect venomous snakes over other snakes or snake-like animals like worms. In fact, several previous studies (Cave and Batty, 2006; LoBue et al., 2010; LoBue and Rakison, 2013; LoBue, 2014) claimed that low-level perceptual features commonly found in threatening stimuli might be detected very quickly by the human visual system. Nonetheless, we should also note that a long history of primate-snake competition could create a particular evolutionary environment unlike the ones existing with other animals (see Isbell, 2006, 2009). In this case, it is also possible that the aposematic coloration (Poulton, 1890) found in some snakes might have had a role inducing avoidance from humans as snakes predators, signaling their **noxiousness**, particularly in large size snakes (Niskanen and Mappes, 2005) more unable to be cryptic. Albeit fear of snakes does not seem to be influenced by aposematic coloration (Prokop et al., 2018), it does increase human attention, although experimental work by Niskanen and Mappes (2005) has shown that at least some snake color patterns may be aposematic without strongly reducing crypsis.

This review also noticed that snake bites are mainly due to the lack of early visual contact with the snake, and only some bites occur during clear visual contact, when the victim is aware of the snake and deliberately handling the animal (e.g., Minton, 1996; Chippaux, 1998; Hossain et al., 2010). It seems that a fast detection mechanism is only useful at a certain distance, and it would be important to know that distance. Predators can attack rapidly leading the potential prey to evolve anti-predator behavior that allows avoiding them (Clark et al., 2012). Nonetheless, the fact that parasympathetic activation—freezing—could also be an automatic and adaptive response (Campbell et al., 1997) to evolutionary threatening stimuli should be noted. Fear bradycardia—slow heart rate—is considered an adaptive response to predators when they are about to attack because the lack of movement may prevent the animal to carry out the intended action.

Some characteristics of human-snake interaction presented here could be argued as favoring the snake detection theory. As snakes are more likely to bite when people are sleeping, or when they are moving about in obscuring vegetation, or when light conditions are low can be seen as evidence that people are bitten because they had no chance to detect the snake. Thus, a modular theory would claim that a non-conscious, fast visual detection, involving the superior colliculus and pulvinar, should allow us to suddenly stop in our tracks before we take a fatal last step. It is not a conscious action, but one that keeps us safe until the slower conscious visual system will enable to actually become aware of the snake. Considering the lack of “sharpness” relative to the first fast visual detection could make us too prone to false positive alarms, and anything similar to a snake would trigger a false alarm. Nonetheless, most situations in which snakes bite people suggest either a total impossibility of escape from the bite—e.g., while sleeping, poor visibility at night, or accidentally stepping a snake while working—or lack of care. If the person is too close when detects the snake, and it is already initiating a strike, there is no likelihood of escape. In contrast, if the snake is detected before one reaches a dangerous distance, approximately 60 cm, speed advantage is no longer necessary, since most snakes will not approach to bite humans.

In addition, Landová et al. (2018) recently noted that agreement in the evaluation of snake fear and also its beauty is cross-culturally high. They also found that the relative fear attributed to a selected snake species is not directly explainable by the current environmental and cultural differences (the Czech Republic and Azerbaijan) providing some support for the evolutionary hypothesis of preparedness to fear snakes.

It seems that more evidence is required to discard or prove the snake detection theory and to support alternative theories, and further studies are required to disprove or prove the snake detection theory. Albeit there is not yet clear evidence for a functional subcortical route for visual processing in primates (Pessoa and Adolphs, 2010), we also assume the possible existence of both innate and acquired learning processes in fear acquisition to snakes among primates in general. For example, Meno et al. (2013) noticed that white-faced capuchin monkeys (*Cebus capucinus*) infants as young as 1 month of age to call alarm at live snakes, which might reflect innate perceptual processes, but there is later in development more detailed predator-recognition processes that require experiential and vicariant learning. Another example is the case of avian predators, innately programmed to flee when in startling proximity to something that resembles another species eye. But these eyes patterns do not exactly mimic any particular predator. Reactions vary in connection with the bird’s learning ability, personal history of predator avoidance and experience, light level, proximity, degree of obstruction or hunger (Janzen et al., 2010; see also Hossie et al., 2015).

Taken together, we would like to draw attention to a potential bias caused by overreliance in controlled laboratory paradigms without naturalistic supportive feedback. Experiments using very simple schematic stimuli in order to control for low-level features are likely to lose some real value and should be confronted with ethobehavioral studies. Ecologically more valid studies could provide a way to study innate and learned fear interactions, and functional relationships in situations likely to occur in the wild (Pellman and Kim, 2016). Furthermore, theories that rely on general feature detection rather than detecting specific species seem more credible from an evolutionary psychology perspective.

## Author Contributions

CC and PS wrote the first draft of the manuscript. AF, FF-S, and AZ wrote sections of the manuscript. All authors contributed to the conception and design of the study and contributed to the manuscript revision, read, and approved the submitted version.

### Conflict of Interest Statement

The authors declare that the research was conducted in the absence of any commercial or financial relationships that could be construed as a potential conflict of interest.
